# The Effect of a Statewide Policy on High School Emergency Action Plans

**DOI:** 10.3390/sports10100161

**Published:** 2022-10-20

**Authors:** Samuel T. Johnson, Michael C. Koester, Viktor E. Bovbjerg, Marc F. Norcross

**Affiliations:** 1 College of Public Health and Human Sciences, Oregon State University, Corvallis, OR 97330, USA; 2 Slocum Center for Orthopedics and Sports Medicine, Eugene, OR 97401, USA

**Keywords:** emergency preparedness, policy development, best-practice guidelines

## Abstract

Institutions sponsoring athletics must be prepared for emergencies. Due to this, more governing bodies are requiring a sports-related emergency action plan (EAP). Yet, the effects of these policies are unknown. We compared adoption of EAPs and associated best practices in Oregon high schools before and after a policy requiring an EAP. Athletic directors were invited to complete a survey during the year before the policy went into effect and again the following year. We assessed whether the school had a written EAP and if they did, was the EAP venue specific, available at the venue, distributed to personnel, and annually reviewed and rehearsed. Pre/post-policy proportions were analyzed using Fisher exact tests for all schools and then schools that completed both surveys. There was a significant increase of schools that reported having an EAP after the policy went into effect (all schools: 55% to 99% [*p* < 0.001] and schools responding both years: 60% to 98% [*p* < 0.001]). Venue specific EAPs also significantly increased but only when analyzing all responses (59% to 71% [*p* = 0.03]). No best practice recommendations related to EAP availability, distribution, review, or rehearsal changed after the policy. Schools met the minimum requirements of the policy, but other related best practices did not significantly improve.

## 1. Introduction

Even though emergencies are relatively rare in secondary school athletics, given the criticality of these situations, an appropriate initial response is crucial in increasing the likelihood of a positive outcome. A fundamental component of emergency preparedness is an emergency action plan (EAP), a written document that defines the actions and care to be provided during an emergency [1,2]. While having a written EAP is an important first step, it is essential that first responders—including school personnel such as coaches, athletic directors (ADs), and athletic trainers (ATs)—know the plan and be prepared to carry it out [1,2].

Multiple organizations have published best practice recommendations advocating that all institutions sponsoring athletics have EAPs that are distributed to all athletics personnel, readily available at all venues, and reviewed and rehearsed annually [1,2]. Despite this, not all secondary schools have an athletic specific EAP [3,4,5] and if they do, the most likely initial responders (e.g., coaches, school officials, ATs) may not have access to it and/or may not have practiced it [4,5].

Several states have enacted laws requiring athletic specific EAPs in secondary schools [6] and some secondary school sports governing bodies have policies requiring EAPs. For example, the Oregon School Activities Association (OSAA) instituted a policy effective August 2016 that, “Each full member school shall have an Emergency Action Plan (EAP) in place for responding to life-threatening emergencies in after school practices and events” [7].

Despite the increased attention and policies related to emergency preparedness in secondary school athletics, to our knowledge, there has been no examination of whether such policies influence EAP adoption and other best practice recommendations related to emergency preparedness in secondary schools. Specifically, we were interested to know if high school ADs were more likely to report the presence of a written EAP for sports-related emergencies after the policy requiring one in Oregon high schools went into effect. Additionally, we investigated associated best practice recommendations related to EAPs, including whether there was an increase in ADs reporting the EAP was venue-specific, distributed to athletics personnel, available at each venue, and reviewed and rehearsed annually—all of which are components that could enhance EAP effectiveness.

## 2. Materials and Methods

The OSAA provided the research team with the names and email addresses of ADs at OSAA full member schools in the spring before the policy went into effect for the following academic year and again the following spring (the academic year the policy went into effect). We invited the ADs of the 285 schools to complete an online survey (Qualtrics, Provo, Utah) regarding their school’s emergency planning.

### 2.1. Instrumentation

We developed the survey based on published best-practice recommendations that all secondary schools have a venue specific EAP that is available (posted) at all venues, distributed to athletics personnel, and reviewed and rehearsed at least annually [1,2]. The survey was identical both years except that the post-policy survey had an additional response choice of “Unknown” for the questions about the frequency of EAP review and rehearsal.

The survey logic was designed so all respondents were asked whether their school had a written EAP for responding to life-threatening emergencies during after-school practices and events. If the AD reported the school did not have an EAP, they were not asked any additional EAP questions. If the AD reported the school had an EAP, they were asked subsequent questions related to the EAP. (See Table 1 for survey questions.).

### 2.2. Data Analysis

We analyzed the effect of the policy on presence of an EAP and other EAP best practices by first examining all schools and then examining schools with responses for both the pre- and post-policy surveys. We created 2 × 2 contingency tables for each of the best practice recommendations we investigated.

The questions about EAP location, EAP review, and EAP rehearsal were multiple response or had multiple answers that could be considered to meet best practice recommendations. Therefore, best practice was defined as follows to create 2 × 2 contingency tables. For EAP location, best practice is to have the EAP(s) posted at all venues [2]. For EAP review and rehearsal, it is recommended that both occur at least annually [1,2]. Consequently, the AD had to answer “Multiple times a year” or “Once a year” to be considered to have met best practice for review and rehearsal. For the post-policy survey, “Unknown” was pooled with the schools that did not review or rehearse the EAP at least annually

Proportions are presented with 95 percent confidence intervals (CI). The 2 × 2 contingency tables were analyzed using Fisher exact tests (alpha ≤ 0.05). Data were analyzed using RStudio version 1.1.463 (RStudio, Inc. Boston, MA) using the tidyverse package version 1.2.1.

## 3. Results

The response rate for the pre-policy survey was 51% (*n* = 144) and post-policy was 25% (*n* = 70). There were 47 schools that responded to the survey both before and after the policy went into effect.

Surveys with incomplete responses had missing data deleted pairwise, except for three questions. First, three ADs (pre-policy *n* = 1; post-policy *n* = 2) did not answer if their school had a written EAP, but all three reported they had venue specific EAPs. Based on the logic that venue specific EAPs require a written EAP, the missing data for the written EAP was imputed to “Yes.” Second, two ADs (one in each year) did not answer whether the school had venue specific EAPs. However, both reported the school’s EAPs were in the school/AD’s office, while one also noted the EAPs were posted at some of the venues and that “each coach is given a copy for his or her area.” Since neither reported the EAPs were posted at all athletic venues, the missing values were imputed to “No”—the schools did not have venue specific EAPs. Third, two schools in the pre-policy survey selected the “Other—please specify” response for location of the EAPs and one reported the “school office” and one reported the “front office,” even though “In the school/AD’s office(s)” was a response choice. Those two responses were added to the tally of responses for school/AD’s office.

At the end of the year that the policy requiring an EAP went into effect, nearly all the schools reported having one, which was significantly greater than immediately before the policy (Table 2 and Table 3). There was also an increase in the proportion of schools reporting venue specific EAPs following the policy; however, the increase was not statistically significant for schools that responded to the survey both years.

Very few schools reported having the EAPs available at all venues both before or after the policy. In contrast, a majority of the ADs reported distributing the EAP to all personnel with a role in carrying out plan—both before and after the policy went into effect. Likewise, a majority of the schools reported reviewing the EAP at least annually. Lastly, a little under half of the schools reported rehearsing the EAP at least annually after the policy, which was not statistically different from before the policy.

## 4. Discussion

Our study is the first to report on the impact of a policy requiring EAPs for athletic-related emergencies in secondary schools. We found that after the policy went into effect there was a significant increase in ADs reporting their school had a written EAP. In contrast, only one of the other five associated best practices for emergency preparedness increased following the policy going into effect. This one effect was an increase in venue specific EAPs, but the effect was only significant when all school responses were analyzed, not when the analysis only included schools that responded to both the pre- and post-policy surveys.

The most promising finding was that nearly all ADs reported their school had a written EAP for athletic related emergencies. There was an almost 40 percentage point increase from the year before the policy went into effect to the year that an EAP was required. The finding that nearly all schools had an EAP, is similar to a report that 95% of ADs in Arizona high schools reported having an EAP [4]. The results from that study and our study are greater than the 53–75% reported in previous studies that asked ADs about the presence of EAPs at their schools [8,9,10,11,12]. Interestingly, the high school athletics associations in Oregon and Arizona used different approaches to increase EAP adoption. In Oregon, every member school of the OSAA is required to have an EAP. In Arizona, schools must have a written EAP to host a postseason contest or event [13]. Additionally, the Arizona Interscholastic Association created pre-contest medical preparedness cards with four questions related to medical emergencies that were distributed to officials and coaches with the expectation that the questions were answered before every contest [14]. The card included the question, “Is there an Emergency Action Plan (EAP) in place?” To our knowledge the effects of the pre-contest preparedness cards has not been evaluated, but it could be surmised that the cards raised awareness of the need for an EAP. Due to the different approaches, it is difficult to determine the primary driver(s) that resulted in nearly all schools in both states reporting they had an EAP.

There was also a significant increase in venue specific EAPs, which is an important consideration due to the unique aspects of each athletic facility. However, this approximate 20 percentage point increase was only statistically significant when all participating schools were examined—not when the analysis was limited to only schools that completed both the pre- and post-policy surveys. Encouragingly, approximately three-quarters of the schools reported having venue-specific EAPs the year the policy went into effect. This is nearly the same as the recent study of Arizona high schools [4]. However, it is greater than a study of Oregon high schools conducted three years before the EAP policy went into effect, which reported only 38% of high schools had venue specific EAPs [3]. Taken together, there has been a gradual increase in venue specific EAPs in Oregon. The reasons for this increase are unknown but it is unlikely due solely to the new EAP policy. While this increase is positive, approximately a quarter of the schools did not report having venue specific EAPs. Understanding the barriers and facilitators to venue specific EAPs is needed to help the remaining schools to develop venue specific EAPs.

No other best practice recommendations for emergency preparedness changed after the policy. These results raise questions regarding the quality of the EAPs that have been developed by the schools and the preparation of the initial responders to carry out the EAP. As previously noted, beyond having the written EAP, the initial responders need to know the plan and be prepared to carry it out. Specifically, the schools should ensure the EAP is available to potential responders and reviewed and rehearsed at least annually.

Best practice recommendations state that the EAP should be distributed to all personnel with a defined role in executing the plan and a hard copy of the document be available at each venue [1,2]. The results of the post-policy survey did not indicate improvement in either practice. Most ADs responded that the EAP was distributed, but very few noted it was posted at each venue. This is an interesting discrepancy that can be potentially explained by several factors. Foremost, it is easy to distribute the EAP to coaches and other potential initial responders. Second, we have heard anecdotally from some school administrators that since non-school groups use the school’s athletic venues and the EAPs are specific to school personnel, they are hesitant to post the EAP due to medico-legal concerns. This raises the question as to what the most effective way is to ensure the initial responders have access to the EAP—distribution, posting, or potentially a different approach.

Ensuring that personnel are appropriately prepared to carry out the EAP is an important element of emergency preparedness. Therefore, review and rehearsal of an EAP is necessary. Approximately three-quarters of participating Oregon ADs reported reviewing the EAP at least annually, which is similar to the recent study of Arizona high schools [4]. However, fewer than half of the schools in our study reported that the EAP was rehearsed at least annually. These results are slightly higher than previous studies of ADs, which found that 18–37% of schools rehearse the EAP at least annually [4,10,11,12]. The difference between review and rehearsal makes sense. Review was defined in the survey as evaluating and updating the plan as necessary, whereas rehearsal was defined as practicing the plan. It can be assumed that it is easier for school personnel to update (i.e., review) the EAP yearly than it is to organize practices with all potential responders (i.e., rehearse). However, both are important elements of emergency preparedness and a better understanding of the barriers and facilitators to each is needed.

There are several limitations to our study. We asked ADs to self-report on the availability, distribution, review, and rehearsal of EAPs instead of directly evaluating the school’s EAPs to determine whether the EAPs met best practice recommendations. The survey questions were based on published best practice recommendations and an underlying assumption of the study was that ADs were honest in their responses. To investigate the accuracy of the responses, we performed a secondary data analysis. In addition to sending the survey to ADs, the same survey was simultaneously sent to the school’s AT if the school had an AT available. As the primary health care provider for sports-related injuries that occur at the school, the AT is responsible for providing emergency care and is trained in developing and implementing EAPs. Unfortunately, only about half of Oregon high schools have an AT available [15]. There were 50 schools over the two surveys where both the AD and AT responded, with a 92% agreement regarding having a written EAP and 83% agreement that the EAPs were venue specific. The level of agreement for meeting best practices for availability, distribution, rehearsal, and review were 81, 62, 55, and 76% respectively. It is not unsurprising that the level of agreement for these EAP best practices were lower because they require more communication between the AD and the AT. For example, an AD may distribute the EAP to all coaches, but the AT may be unaware of it, or the AT may organize a rehearsal of the EAP, but the AD is not told. While we believe this communication should be occurring it is plausible to see why it may not occur.

Another limitation was the low response rate to the post-survey (25%). We do not know why that response rate was half of the pre-survey rate. Despite this, we believe the results of the study are useful. It is reasonable to assume that the ADs that responded were either (1) representative of all ADs in the state or 2) more interested in the topic such that their behaviors are expected to be more consistent with best practices. Therefore, if the low response rate resulted in a biased sample (i.e., assumption number (2), we would expect the findings would be an overestimate of the true state of best practice adoption. Considering that except for an increase in written EAPs and venue specific EAPs (when examining the entire sample), the other EAP best practices we studied did not change the year the policy went into effect. If anything, the adoption of best practices would be worse than the findings suggest, further enforcing our call for improvements in meeting best practice recommendations.

## 5. Conclusions

Our results indicate that the vast majority of responding Oregon schools are meeting the minimum of the policy—having an EAP for athletic-related emergencies available at the school. This appears to be an important initial step [16]. However, almost all of the other best practice recommendations—ones that likely facilitate better care in an emergency—were still lacking. This raises fundamental questions on how to improve the EAPs to ensure proper care during an emergency. Is more education and awareness of the importance of EAPs needed? Are more detailed policies and procedures required? Are different approaches needed to help the schools? Future research is necessary to elucidate what approach or combination of approaches are needed. Until then, there are a variety of approaches that may be helpful to both the governing bodies of secondary school sports and schools. The governing bodies could bolster policies by stipulating the EAPs include specific components outlined in the best practice guidelines. They could also provide education and assistance to schools and districts on how to develop and implement EAPs that meet best practice guidelines. Our results indicate the biggest gaps in meeting best practices are failure to rehearse the EAP regularly or posting the EAP at the venue. School administrators should work with coaches and other athletic personnel to require realistic practice in carrying out the actions detailed in the EAP. Additionally, school personnel should work collaboratively on the best way to ensure all local first responders have access to the EAP—whether that is through distribution to all potential first responders or posting of the EAP.

## Figures and Tables

**Table 1:** Survey Questions.

Question Possible answers
1. School name:
2. Does your school have a written emergency action plan (EAP) in place for responding to life-threatening emergencies in after-school practices and events?
o Yes o No
*If answer is No—stop. If the answer is Yes—continue to question 3.*
3. Does your school have written emergency action plans that are specific to each athletic venue? (That is, are there separate emergency action plans for every athletic venue such as the main gymnasium, football stadium, weight room, athletic training room, etc.?)
o Yes o No
4. Where is your school’s emergency action plan(s) located? (Mark all that apply.)
□ In the school/AD’s office(s) □ In the coaches’ office(s) □ In the athletic training room □ Posted at all or some athletic venues □ Posted at all athletic venues □ Others—please specify
5. Is the emergency action plan(s) distributed to all individuals at your school with a defined role in executing the plan—including coaches, administrators, athletic trainers, etc.?
o Yes o No
6. How often is the emergency action plan rehearsed? (That is, practicing the plan in the absence of an actual emergency event.)
o Multiple times a year o Once a year o Once every other year o Never o Unknown *
7. How often is the emergency action plan reviewed? (That is, evaluating and updating the plan as necessary.)
o Multiple times a year o Once a year o Once every other year o Never o Unknown *

* Answer included only on the post-survey.

**Table 2:** Athletic director responses to EAP availability, distribution, review, and rehearsal at *all* schools responding to the survey. Proportions, 95% confidence intervals, and *p*-values from Fisher exact tests are presented. (Pre-policy *n* = 144, post-policy *n* = 70).

Athletic Directors Responding Their School:	Pre-Policy	Post-Policy	*p*-Value
Had a written EAP	55% (46–63%)	99% (92–100%)	<0.001
Had venue specific EAPs	59% (47–70%)	77% (65–87%)	0.031
Had the EAP posted at all venues	7% (2–15%)	11% (4–21%)	0.055
Distributed the EAP to school personnel with a role in carrying out the EAP	70% (59–80%)	67% (54–78%)	0.717
Reviewed the EAP at least annually	80% (69–88%)	73% (60–83%)	0.425
Rehearsed the EAP at least annually	49% (37–61%)	41% (29–54%)	0.398

**Table 3:** Athletic director responses to EAP availability, distribution, review, and rehearsal *at schools responding to the survey both before and after enactment of the policy*. Proportions, 95% confidence intervals, and *p*-values from Fisher exact tests are presented. (*n* = 47).

Athletic Directors Responding Their School:	Pre-Policy	Post-Policy	*p*-Value
Had a written EAP	60% (44–74%)	98% (88–100%)	<0.001
Had venue specific EAPs	54% (34–72%)	71% (56–84%)	0.141
Had the EAP posted at all venues	0% (0–8%)	4% (1–15%)	0.525
Distributed the EAP to school personnel with a role in carrying out the EAP	75% (55–89%)	71% (56–84%)	0.792
Reviewed the EAP at least annually	79% (59–92%)	73% (58–85%)	0.781
Rehearsed the EAP at least annually	36% (19–56%)	49% (34–64%)	0.335

## Data Availability

Data are not shared due to privacy concerns.
